# Effects and mechanisms of synchronous virtual reality action observation and electrical stimulation on upper extremity motor function and activities of daily living in patients with stroke: a protocol for a randomized controlled trial

**DOI:** 10.3389/fneur.2025.1499178

**Published:** 2025-04-04

**Authors:** Yao Cui, Fang Cong, Ming Zeng, Jun Wang

**Affiliations:** ^1^Department of Physical Therapy, Beijing Bo'ai Hospital, China Rehabilitation Research Center, Beijing, China; ^2^School of Rehabilitation Medicine, Capital Medical University, Beijing, China; ^3^Department of Rehabilitation Medicine, The Second Affiliated Hospital of Jiaxing University, The Second Hospital of Jiaxing City, Jiaxing, Zhejiang, China; ^4^Department of Physical Therapy, Hangzhou Geriatric Hospital, Hangzhou, Zhejiang, China

**Keywords:** mirror neuron system, action observation therapy, virtual reality, electrical stimulation, stroke, rehabilitation, fNIRS, brain plasticity

## Abstract

**Background:**

Existing rehabilitation techniques are not satisfactory in improving motor function after stroke, resulting in heavy social burdens. With discovery of mirror neuron system (MNS), action observation (AO) has become a promising strategy to promote motor learning in rehabilitation. Based on MNS theory and virtual reality (VR) technology, we designed an innovative rehabilitative approach: synchronous 360° VR video AO (VRAO) and neuromuscular electrical stimulation (NMES). We hypothesized that VRAO+NMES could enhance MNS activation, thus to improve upper limb motor function and activities of daily living in stroke survivors.

**Methods:**

To explore the efficacy and mechanism of VRAO+NMES, we designed this single center, evaluator blinded, prospective, two arm parallel group randomized controlled trial with 1:1 allocation ratio. The experiment group will receive VRAO+NMES, while the control group will receive VR landscape observation combined with NMES. The Fugl-Meyer Assessment for Upper Extremity is the primary outcome of this study, Brunstrom Recovery Stages for Upper Extremity, Manual Muscle Test, Range of Motion, Modified Barthel Index, and Functional Independence Measure are the secondary outcomes. In addition, functional near-infrared spectroscopy (fNIRS) and surface electromyography (sEMG) will be used to evaluate the activation of MNS brain regions and related muscles, respectively.

**Discussion:**

Applying VR in AO therapy (AOT) has become popular, another study direction to improve AOT is to combine it with peripheral stimulations simultaneously. Due to its full immersive characteristic and multi-sensory input, 360° videos based VRAO+NMES could improve the motivation and engagement level of participants. In addition, fNIRS and sEMG test results may act as good biomarkers to predict rehabilitation outcomes, helping select suitable candidates for this new intervention.

**Conclusion:**

The results of this study will provide evidence for the feasibility and potential clinical efficacy of VRAO+NMES in stroke rehabilitation, thus to promote the clinical applicability and generalize its use in hospital, community, and home rehabilitation settings.

**Clinical trial registration:**

https://www.chictr.org.cn/showproj.html?proj=178276, Identifier [ChiCTR2200063552].

## 1 Introduction

Stroke is the second-leading cause of mortality and the third-leading cause of disability globally (1). The physical, cognitive, and emotional disorders caused by stroke influence the patients’ life comprehensively and dramatically; approximately half of stroke survivors are chronically disabled and need long-term rehabilitation (2). Promoting changes in brain structural plasticity and functional reorganization are critical to rehabilitation of motor function following stroke (3). In physiotherapy, motor learning and relearning have become important strategies to induce neuroplasticity (4). Recently, the discovery of mirror neuron system (MNS) has further advanced our understandings of the neuroscientific mechanisms underlying motor learning and brain functional reorganization (5). Mirror neurons are a special class of neuron that excites during both observation and execution of actions. It is, therefore, possible to activate the motor system by simply observing actions without actual motor outputs (6).

The motor system has the potential to learn new skills or to recover from injury by observing others’ actions through activation of MNS (7). Meta analysis results have showed that AO therapy (AOT) is helpful to improve stroke patients’ upper extremity and hand functions, walking ability, and activities of daily living (ADLs); it has been accepted as an effective method in neurorehabilitation (8–10).

With technological advances, two important study directions have recently emerged to further promote motor learning effects induced by activation of MNS. The first direction is to change the implementation methods and techniques of AOT to improve participants’ engagement. The second direction is to combine AOT with other rehabilitation techniques, thus to obtain synergistic effects and improve treatment efficacy (11).

In the first research direction, virtual reality (VR) technology has become increasingly important. As a new modality of rehabilitative approach, VR can offer multisensory integration, including visual, tactile, vestibular, proprioceptive, interoceptive, emotional, and somatosensory inputs, creating a favorable rehabilitation environment (12). According to immersion level, VR can be divided into immersive, semi-immersive, and non-immersive VR (13). There are several types of immersive VR, graphical computational three-dimensional (3D) animations VR and 180°/360° videos VR are the most common ones (14). Compared with watching two-dimensional (2D) videos on a computer screen, participants can change their point of view freely to watch the demonstration from different angles wearing a VR headset (15).

With advances in technology, 360° videos based fully immersive VR treatments are becoming more accessible and flexible, playing an important role in enhancing wellbeing (16). The immersive feelings facilitate engagement and motivation of participants; 360° VR videos are good choices to improve immersive feelings (17). With the highest levels of immersion and reality, the novelty of 360° VR videos will make AOT more interesting and motivating. Furthermore, by capturing or displaying a full spherical view, 360° VR provides a more authentic and immersive experience, closely resembling real-life perception (18). As the result, the enhanced immersive feelings will motivate participants to view the videos and try to imitate the actions (19, 20). In addition, the high frame rate (e.g., 100 fps) of the modern 360° camera allows users to capture slow motions of the observed actions, helping to show step-by-step details of daily life actions.

The second research direction involves obtaining synergistic effects through combination of AOT with other rehabilitation techniques. This is mainly based on the theory that central-peripheral synchronous stimulations have the potential to improve brain activity (21). To further evoke plasticity in human motor system, AOT has been reported to be combined with different treatments, including transcranial magnetic stimulation (TMS), electrical stimulation (ES), and rehabilitation robots (11). The most common peripheral stimulation is low-frequency ES, which is a kind of physiotherapy modality used to improve motor and sensory function, including neuromuscular electrical stimulation (NMES), functional electrical stimulation (FES), and peripheral nerve stimulation (PNS) (22). When applying AOT+PNS in healthy individuals, the PNS-generated afferent signals from the periphery induce neural plasticity in primary motor cortex (M1) (23). Seitz et al. (24) studied the immediate effect of concurrent applications of AO, motor imagery (MI), and PNS, showing that AO+MI+PNS can induce plasticity in M1.

Based on the above background, we designed a new kind of rehabilitative technique: concurrent VR-based AO combine with NMES (VRAO+NMES). By playing immersive 360° videos in a VR headset, we present visual information to participants. We have tested the validity of combination of AO and NMES in a previous study, using 2D video clips of hand extension movements as the modality of AOT. We tested brain activation patterns of left MNS during AO, action execution, and imitation combined with NMES in healthy participants, the results proved that MNS-based rehabilitation approaches may enhance cortical activation of MNS (21). In addition, we also confirmed that compared with action execution and 2D video based action imitation, the activation of MNS was enhanced when combing them with NMES (11). Here, we will further test the VRAO+NMES application in stroke rehabilitation.

Due to the effects of action visual stimulation on MNS and ES on motor units (MUs), VRAO+NMES may be helpful to accelerate the process of motor learning. In addition, the enhanced multisensory interactions generated by VRAO+NMES have potential to improve the alteration of body ownership and induce self-body recognition (23). To investigate the potential neuromuscular control mechanism underlying this new intervention, we will use functional near-infrared spectroscopy (fNIRS) and surface electromyography (sEMG) to evaluate the activation of brain regions and muscles, respectively. In addition, MNS network-based neuroplasticity changes have been confirmed recently, we will also explore the brain functional connection changes before and after the interventions (25).

We, therefore, aim to determine whether a concurrent application of VRAO and NMES would result in significant improvements in behavioral assessments and neurophysiological parameters, and to test whether the changes in motor function evaluated by clinical scales are related to the brain functional imaging results measured by fNIRS. We hypothesize that VRAO+NMES is superior in improving upper extremity and hand motor functions, ADLs, and MNS cortical activation levels compared to conventional therapy plus NMES. The underlying mechanism may be that the brain-muscle synchronous intervention enhances activation of the MNS and recruitment of MUs, thereby promoting brain plasticity changes, improving neuromuscular control function, and enhancing therapeutic effects.

## 2 Materials and methods

### 2.1 Study design and settings

**Figure 1:**
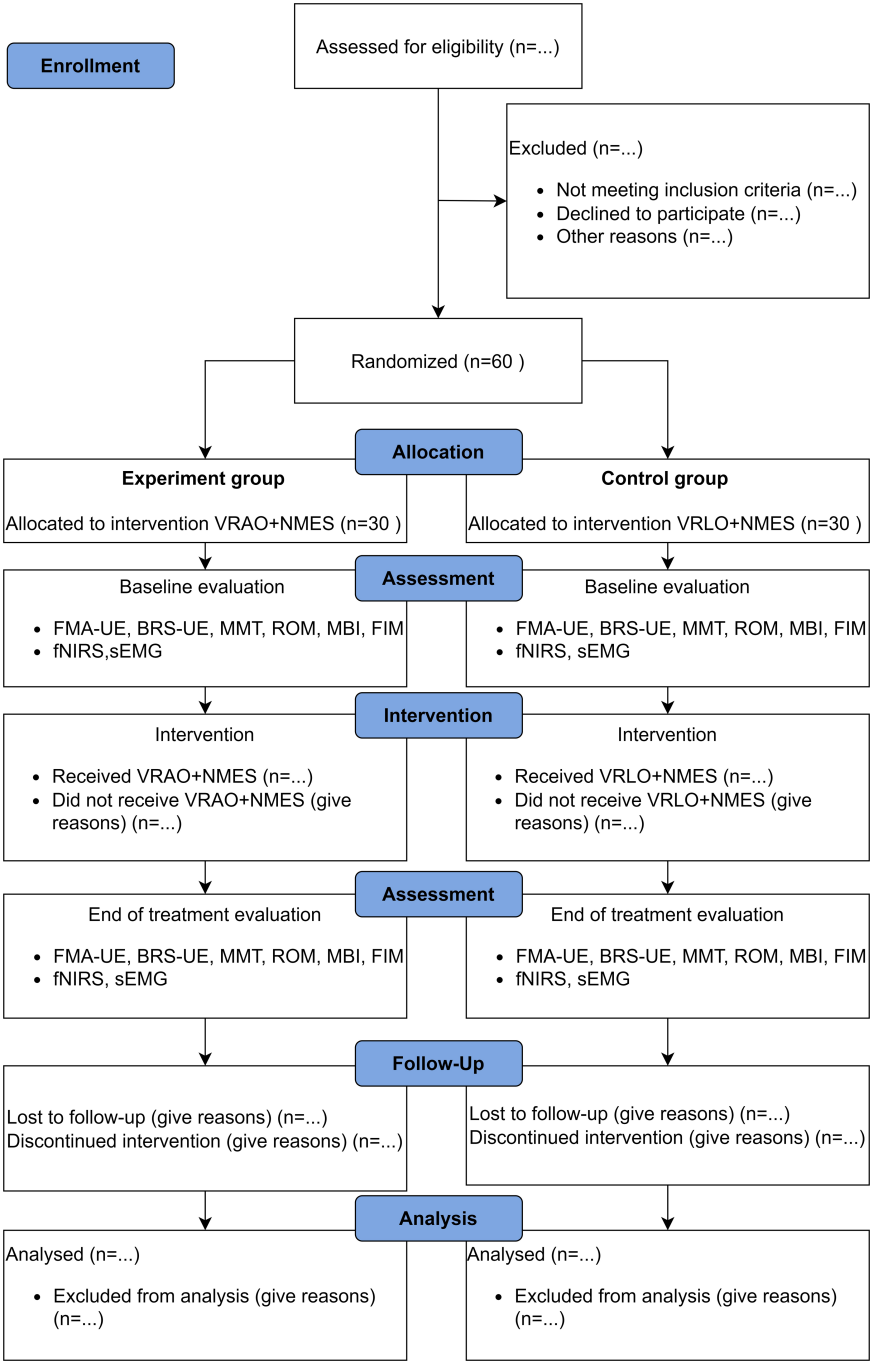
Flowchart of study design according to CONSORT 2010. VR, virtual reality; AO, action observation; LO, landscape observation; NMES, neuromuscular electrical stimulation; FMA-UE, Fugl-Meyer Assessment for Upper Extremity; BRS-UE, Brunnstrom Recovery Stages for Upper Extremity; MMT, Manual Muscle Test; ROM, Range of Motion; MBI, Modified Barthel Index; FIM, Functional Independence Measure; fNIRS, functional near-infrared spectroscopy; sEMG, surface electromyography.

This study is a single center, evaluator blinded, prospective, two arm parallel group design randomized controlled trial (RCT) with a 1:1 allocation ratio (Figure 1). This trial protocol adheres to the Standard Protocol Items: Recommendations for Interventional Trial (SPIRIT) statement, and the SPIRIT 2013 checklist (26). The study results will be reported following the Consolidated Standards of Reporting Trials (CONSORT) statement and checked with the CONSORT 2010 checklist (27). The study will comply with the principles of Good Clinical Practice, and the Declaration of Helsinki and its amendments.

Each participant will voluntarily sign a written informed consent prior to inclusion. This study will be conducted at Beijing Bo’ai Hospital, China Rehabilitation Research Center (CRRC). This study was approved by the Medical Ethics Committee of CRRC (approval number: 2021-100-1). This study was registered in the China Clinical Trial Registration Center (Registration number: ChiCTR2200063552) and the Medical Research Registration Information System of the National Health Security Information Platform (Registration number: MR-11-23-018905).

### 2.2 Participant timeline

**Figure 2:**
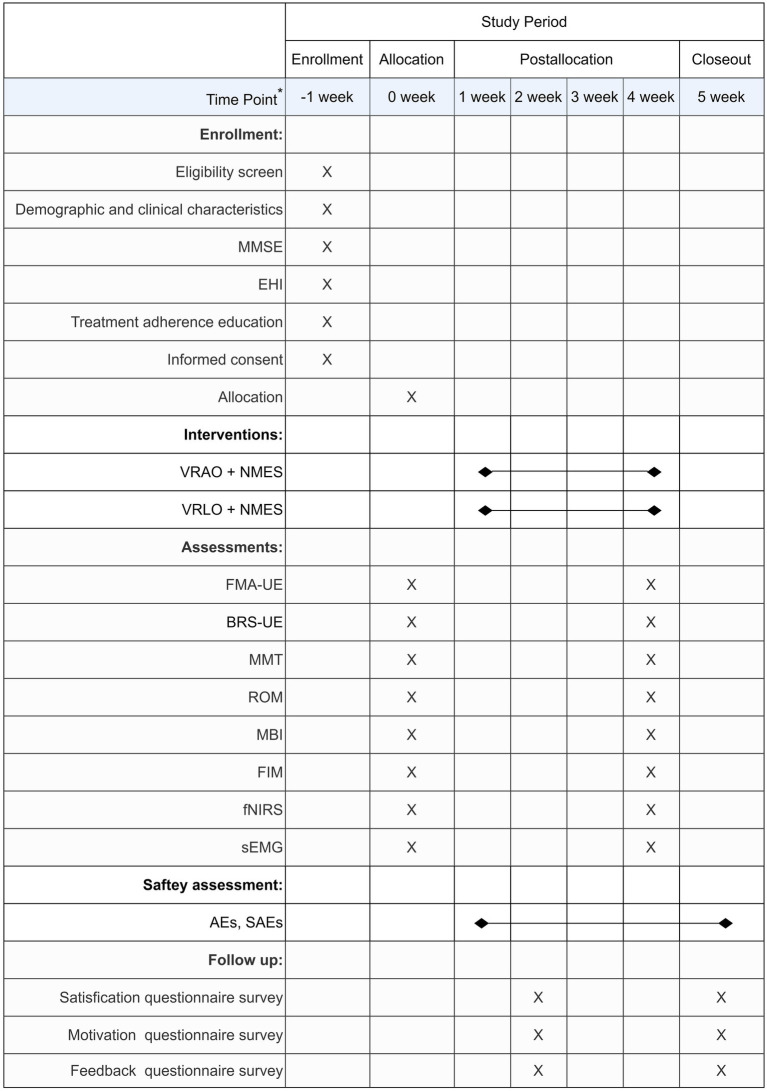
Timeline of participant enrolment, allocation and analysis according to SIPIRIT 2013. VR, virtual reality; AO, action observation; LO, landscape observation; NMES, neuromuscular electrical stimulation; FMA-UE, Fugl-Meyer Assessment for Upper Extremity; BRS-UE, Brunnstrom Recovery Stages for Upper Extremity; MMT, Manual Muscle Test; ROM, Range of Motion; MBI, Modified Barthel Index; FIM, Functional Independence Measure; fNIRS, functional near-infrared spectroscopy; sEMG, surface electromyography; AEs, adverse events; SAEs, serious adverse events.

The schedule of enrollment, allocation, interventions, and assessments of this study is following the SPIRIT statement (Figure 2). A follow-up survey will be conducted at 1 week after the end of the trial, including questionnaire surveys of safety, satisfaction, motivation, and feedback.

### 2.3 Screening and recruitment

Patients with stroke will be recruited and screened for eligibility at Beijing Bo’ai Hospital. The study introduction poster and flyers will be distributed to potential participants. Detailed study information will be provided to all the intended participants and their caregivers by a trained researcher during the screening phase. Participants can contact the research coordinator to acquire more study information during their decision-making process. Recruitment stops once the pre-specified sample size is reached, or the preplanned interim analyses show the new intervention is unlikely to demonstrate benefit. All decisions will be reviewed by the Data Safety Monitoring Board and the Medical Ethics Committee.

### 2.4 Participants

Patients with stroke will be recruited according to the following criteria.

#### 2.4.1 Inclusion criteria

Participants will be included if they meet the following criteria: (1) Patients with a first-ever ischemic or hemorrhagic stroke, meeting the stroke diagnostic criteria established by the fourth National Conference on Cerebrovascular Disease of China in 1995, and confirmed by computerized tomography (CT) or magnetic resonance imaging (MRI); (2) Adults, aged 30–70 years old; (3) Within 6 months of stroke onset; (4) The disease is stable, neurological conditions and vital signs are no longer progressing; (5) The ability to sit independently for approximately 40 min to finish the treatments and assessments, including preparing time; (6) Without visual impairment, able to see the content of the experiment program; (7) Unilateral limb hemiplegia, Brunstrom Recovery Stages for Upper Extremity 3, 4, or 5 stage at the time of enrollment; (8) Right-handed, determined by the Edinburgh Handedness Inventory (EHI) – Short Form (28); (9) Chinese speaking; (10) Junior high school education or above; and (11) Willingness to provide informed consent and comply with the study protocol.

#### 2.4.2 Exclusion criteria

Participants will be excluded if they meet one of the following criteria: (1) With serious diseases, such as heart, lung, liver, and kidney failure; (2) With other neurological conditions that cause motor deficits, such as Parkinson’s disease, multiple sclerosis, and peripheral neuropathy; (3) Epileptic seizures; (4) With cognitive impairments, Mini-Mental State Examination (MMSE) ≤24 scores, unable to cooperate with researchers to finish the interventions or assessments; (5) With severe aphasia and communication difficulties that may influence the intervention and outcome measures; (6) With severe pain, Visual Analog Scale (VAS) ≥7 scores; (7) With moderate or severe depression, anxiety and other psychological disorders; (8) With contagious conditions or diseases, particularly of eyes, skin, scalp, or forearm; (9) With implanted medical devices or materials, such as pacemaker and metal implants; (10) With contraindications for NMES, such as skin injuries or skin diseases, acute infections, vascular diseases in the affected forearm; (11) With other diseases limiting upper extremity and hand functions, such as fracture, joint injury, and muscle pain of the affected arm; (12) With vestibular diseases, digestive problems that limit the use of VR headset; (13) Surgery and other invasive treatments; (14) Participants with a recurrent stroke or any medical condition that may affect safety or accuracy of evaluation results will also be excluded (29).

In addition, we will use structural MRI to calculate lesion volumes, including hemorrhage size (for hemorrhagic strokes) and infarct volume (for ischemic strokes). Automated or semi-automated segmentation tools (e.g., MRIcron, ITK-SNAP) will be applied to ensure accuracy and reproducibility (30). Lesion locations will be mapped to standardized brain atlases [e.g., Montreal Neurological Institute (MNI) space] using voxel-based or region-of-interest approaches. This will allow precise documentation of cortical/subcortical involvement (e.g., motor cortex, corticospinal tract, parietal regions) (31). Participants will be stratified into subgroups based on stroke type, lesion volume and location. What’s more, we will meticulously record the time elapsed from stroke onset to the initiation of rehabilitation, including the start of conventional rehabilitation therapy and AOT.

### 2.5 Randomization and blinding

Through a computerized block randomization protocol, the participants meeting the eligibility criteria will be randomly assigned to control group or experiment group. The random allocation sequence will be generated by the R package blockrand version 1.5, with randomly ordered blocks of size 6 or 8, and a 1:1 allocation ratio. The randomization cards generated by the blockrand version 1.5 package will be printed out and enclosed in sequentially numbered, opaque, sealed, and stapled envelopes. The allocation sequence will be concealed from the researcher responsible for enrolling and evaluating participants. Corresponding envelopes will be opened only after the enrolled participants have completed all the baseline assessments and at the time of allocation to the intervention. An independent study coordinator who will not oversee assessments and interventions will design and conduct the randomization sequence generation and group allocation. Only the study coordinator and principal investigator (PI) have access to group assignment materials locked in a binder.

Due to the nature of rehabilitative interventions, it is impossible to blind participants, their caregivers, and therapists participating in the treatment. In this study, the assessor and data analyst will be blinded throughout the trial. The participants will be instructed not to disclose their allocation to investigators or outcome assessors. The therapists who conduct the treatment will be asked not to disclose the participant’s group assignment during the whole study period (12). In addition, they will not evaluate the participants and have access to the evaluation results (29). To mitigate the potential performance bias due to the lack of participant/therapist blinding, we will take the following actions. Firstly, we will make standard operating procedures (SOPs) for all the assessments and interventions. Adhering to the SOPs will ensure consistency in how the assessments and interventions will be administered across all participants, reducing variability introduced by therapist behavior. Secondly, all the therapists will be trained thoroughly on the SOPs, their adherence throughout the trial will be monitored. Thirdly, we will ensure that the informed consent process does not inadvertently reveal the hypothesized advantages of the experimental intervention. Fourthly, we will actively monitor for signs of performance bias during the trial, such as differential dropout rates or adherence patterns between groups. If participants inadvertently or intentionally reveal treatment details to assessors, we will record all unblinding incidents (timing, cause, and impact) and report them in study publications per CONSORT guidelines.

### 2.6 Intervention

The experiment group will receive VRAO+NMES, while the control group will receive VR landscape observation (VRLO) + NMES; both groups will receive conventional comprehensive rehabilitation therapies and routine medical treatments. During the intervention, the participant will sit upright in a relaxed way, wearing the VR headset and attaching the NMES electrodes. All the intervention sessions will be supervised by certified physiotherapists with more than 5 years of experience. For both groups, the intervention period will last 4 weeks, with a frequency of 5 times a week, and 20 min per session.

To implement the interventions and assessments, we designed a specific VR-based integrated rehabilitation evaluation and training system. The system manages the presentation of VR visual stimulation and the triggers to control the ES output (32). The experiment program adopts block design, the task duration is 15 s and rest duration is 20 s in each block, the electrical output is on during the task period and off during the rest period; this protocol has been confirmed by our previous study (21). Except for contents of the videos, action videos for the experiment group and landscape videos for the control group, all the other parameters are same in both groups. The first-person and third-person point of view 360° VR video clips of actions and landscapes will be shot using an Insta360 one X2 camera, and edited by Insta360 studio v5.2.4 (Insta360 Inc., Shenzhen, China). The videos and experiment cues will be presented through a Oculus Quest 2 VR headset (Meta Platform Inc., CA).

At the beginning of the study, the therapist will explain treatment process to the patient in advance. During each intervention session, the patient will sit in an upright and relaxed position, the therapist will determine the motor points of the target muscles. After disinfecting the skin with medical alcohol, the therapist will stick electrodes and fix cables according to the determined motor points. After putting the VR headset on the participant’s head, the experiment program will be turned on, at this time, the participant could see the visual stimulations and feel the electrical stimulations. A laptop will be linked to the VR headset to display the contents seen by the participant, allowing the therapist to monitor the interventions. During the treatment, the therapist will always be present, observing the patient’s reaction and making treatment records. The participant will be told to stop the intervention at any time if they feel discomfort.

#### 2.6.1 Experiment group

In the experiment group, 360° VR action videos will be played during the NMES output period. During task phase, participants will be guided to view the VR action videos and feel the NMES induced hand movements. During rest phase, they will be encouraged to try their best to imagine or imitate the observed actions. We will build a database of 360° action videos performed by different models with different age ranges, genders, and heights. For each participant, we will choose the most similar model’s action videos according to the participant’s demographic and clinical information, mainly the affected/impaired side, gender, and age. Basic actions of single joints, goal directed actions, and daily life actions will be recorded, including unimanual and bimanual actions. In each treatment session, 4 basic actions and 12 daily life actions will be selected according to the participant’s motor ability. The actions observed in each session will be arranged according to their difficulty and complexity. For each round, a single joint basic movement’s 360° video, such as wrist extension, will be observed first, followed by three daily life actions’ 360° videos, such as picking up objects from the desktop, typing, and holding a pen.

In this study, a customized made electrical stimulation device will be made to conduct the personal treatment protocol: waveform: rectangular; phase duration: 200 μs; pulse frequency: 50 Hz; burst frequency: 1 Hz; ramp up time: 1 s; hold time: 15 s; ramp down time: 1 s; interval time: 18 s. This NMES treatment protocol has been validated by our previous studies (11, 21). The electrical intensity for each patient will be individualized to minimize evoked muscle contractions and avoid discomfort (11).

#### 2.6.2 Control group

As contrast, participants in the control group will watch VR landscape videos during the NMES outputs. The landscape VR videos will be shot by the research team, mainly of nature sceneries of gardens.

### 2.7 Outcomes

All participants will be evaluated by clinical scales, fNIRS, and sEMG before and after 4 weeks of interventions. At baseline, sociodemographic and neurologic data will be collected, including age, gender, Body Mass Index, education, medical history, type of stroke, location of injury, and duration of disease. In addition, questionnaire surveys about safety, satisfaction, and feedback will be performed at the end of the interventions. Adverse events and serious adverse events will be monitored from the beginning to the end of the study. Expert therapists with above 10 years of experience will conduct the functional measures.

#### 2.7.1 Primary outcome

The Fugl-Meyer Assessment for Upper Extremity (FMA-UE) is the primary outcome of this study, which is a 33-item scale used to assess motor function impairment in stroke survivors. Its reliability and validity have been well demonstrated (33). The minimal clinically important difference (MCID) score of FMA-UE is 4.25 scores (34). We hypothesize that more patients will reach a clinically relevant difference of 4.25 scores in the treatment group versus the control group.

#### 2.7.2 Secondary outcomes

Brunstrom Recovery Stages for Upper Extremity will be used to assess severity of the deficits post stroke, it has good reliability and validity (35). Manual Muscle Test will be conducted on the key upper extremity and hand muscles. Passive and active range of motion will be measured of the key upper extremity and hand joints. The Modified Barthel Index is a 10-item scale with good reliability and validity, it is used to assess the patient’s ability to perform ADLs (36). The Functional Independence Measure will be used to measure the ability to carry out everyday tasks safely and independently, thus to determine burden of care, it’s reliability and validity are excellent (37).

#### 2.7.3 fNIRS study

To study brain activation patterns and functional connection changes before and after the interventions, and to compare their difference between the two groups, each enrolled participant will be screened for fNIRS signal quality. Those with good signal quality will be evaluated using fNIRS. The NIRSport2 fNIRS device with 8 sources and 8 detectors, and its supporting data acquisition software Aurora fNIRS version 2021.9.0.6 (NIRx Medical Technologies, MN), will be used. The sampling rate will be set at 10 Hz. The regions of interest are the following Brodmann Areas (BA): BA6, BA7, BA40, BA44, and BA45, mainly the MNS; the montage design and its sensitivity have been confirmed in our previous study (21).

During the screen phase, the signal quality will be evaluated by the Aurora software’s default quality assessment function, it uses signal levels, dark noise and source brightness to reflect the signal quality, the signal level index is selected as the most important signal quality evaluation criterion. Signal level is the raw voltage reading at the detector, it reflects how well light passes through tissue, from a certain source to the detector. If this average value is high enough, the signal level of the channel formed by this source and detector will be marked as excellent (green, > 3 mV) or acceptable (yellow, > 0.5 mV and < 3 mV); if it is too low, it will be marked as critical (red, < 0.5 mV). If 80% of the channels’ signal levels are marked as green, the participant will be chosen to take part in the formal fNIRS assessments.

**Figure 3:**
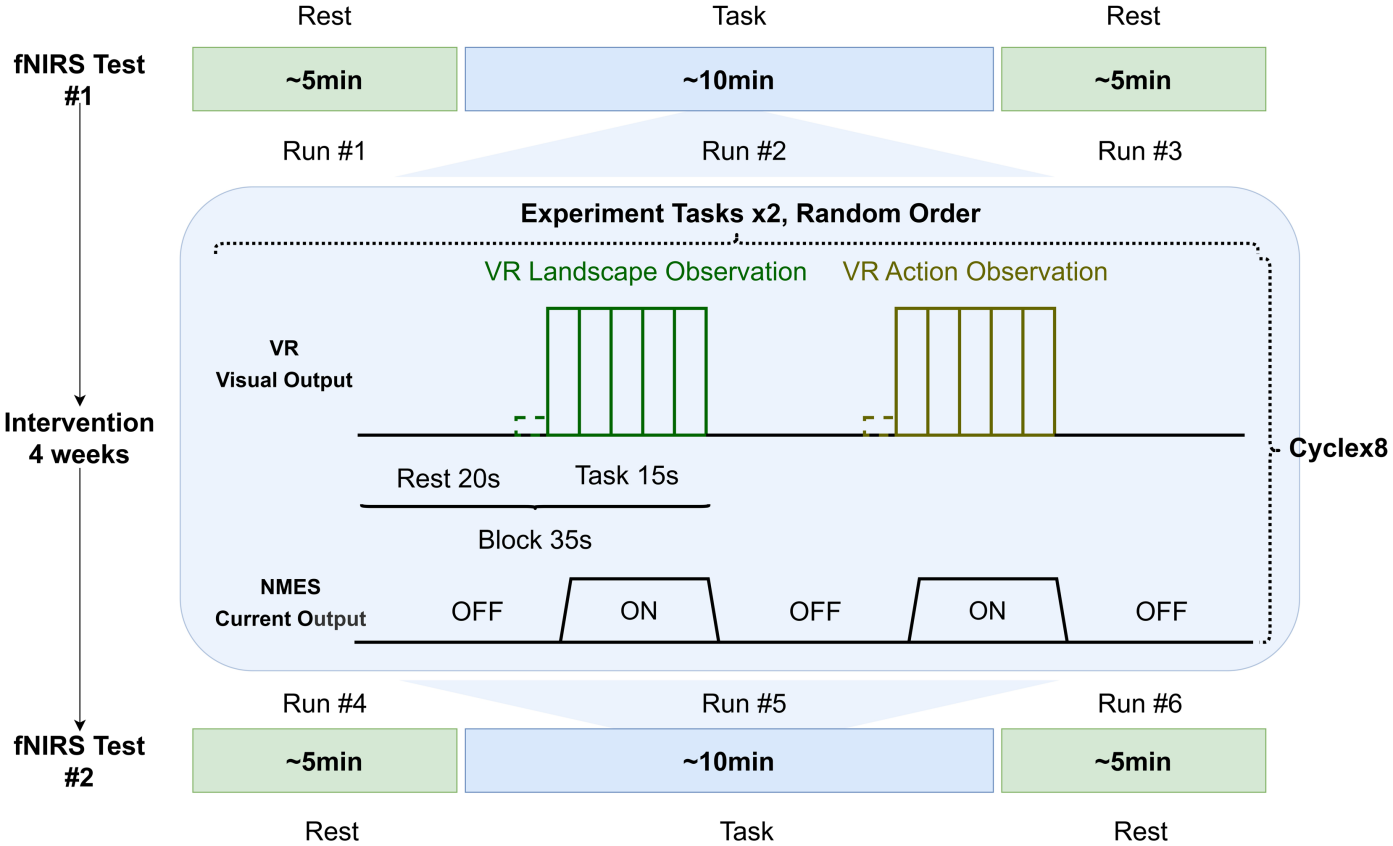
Schematic illustration of the fNIRS experimental paradigm. fNIRS, functional near-infrared spectroscopy; VR, virtual reality.

This fNIRS test protocol is designed to reveal the neural activity changes of a single session and multiple sessions (20 sessions over 4 weeks) of intervention (Figure 3). The test includes two sessions, one before the interventions (0 weeks) and one after the interventions (4 weeks). Three fNIRS testing runs will be conducted in each session: first, ~5 min resting state data will be collected; second, ~10 min task-related data will be collected; third, another ~5 min resting state test will be conducted after finishing the tasks. In each fNIRS test session, participants will seat in a comfortable and relaxed upright position. In the resting state test, participants will be asked to keep still and silent, trying their best to relax as much as possible without thinking about anything, but not falling sleep. The task experimental program adapts a block design with two different conditions: VRAO+NMES and VRLO+NMES. Each block will last 35 s, including 20 s of rest and 15 s of task. Each experimental task will be cycled 8 times. The fNIRS data pre-processing and analysis will be conducted in MatLab R2017b (MathWorks, MA), including individual analysis and group-level analysis (21).

##### 2.7.3.1 Resting-state fNIRS data preprocessing and analysis

The resting-state fNIRS data will be pre-processed by the FC-NIRS Matlab package (38). Firstly, the signal quality will be assessed by the signal-to-noise ratio, between-channel signal correlation, and frequency spectrum analysis. Secondly, the channels with poor quality will be considered as bad channels and removed from analysis. The participants with 5 or more bad channels will be excluded from the analysis. Thirdly, the spline interpolation method will be used to eliminate motion artifacts. Fourthly, the Principal Component Analysis (PCA) algorithm will be used to perform global signal regression to reduce the effect of global noise (arterial pulse, respiration, and cardiac pulsation etc.). Fifthly, the influence of low-frequency drift and high-frequency physiological noise will be reduced by the bandpass filter method with a range of 0.01–0.1 Hz. Sixthly, the hemoglobin (Hb) concentration changes will be calculated according to the modified Beer–Lambert law (mBLL), including oxygenated hemoglobin (HbO) and deoxygenated hemoglobin (HbR). Seventhly, stable Hb time series will be extracted for each participant (39). Finally, the topological properties of the brain network will be calculated based on graph theory, including the global network metrics (clustering coefficient, characteristic path length, global and local efficiency etc.) and nodal network metrics (nodal degree, nodal efficiency, and nodal betweenness) (38).

##### 2.7.3.2 Task fNIRS data preprocessing and analysis

The task related fNIRS data will be analyzed by the NIRS Brain AnalyzIR Toolbox (40). Firstly, the task related fNIRS raw data (light intensity) will be converted into optical density. Secondly, Hb concentration changes will be calculated according to the mBLL (41). Thirdly, the autoregressive iteratively reweighted least squares (AR-IWLS) general linear model (GLM) algorithms will be used to quantitatively analyze the relationship between brain signal and stimulus, the regression coefficient (*β*) will be calculated. Fourthly, the linear mixed-effect model will be used in the group-level analysis, the estimated value of β and its corresponding standard error will be calculated, treating participants as random effects. The false discovery rate (FDR) method will be used to obtain multiple comparison correction *p*-values (P_FDR_) (42). Fifthly, after channel level data analysis, based on the probability registration method, the activation results of each region-of-interest will be obtained. Finally, the brain activation results will be visualized on the Colin27 standard brain (21).

#### 2.7.4 sEMG study

The UMI-SE-I sEMG recording and analysis system (United Medical Equipment Co., Ltd., Shaoxing, China) will be used in the sEMG sub-study. The main parameters of this device are: sampling rate of >3 kHz, resolution of 0.1 μV, passband width of 15–1,000 Hz, common mode rejection ratio of >110 db, and noise of <1 μV. Disposable Ag/AgCl electrodes will be used to collect data after the skin is wiped by 75% medical alcohol. The electrodes will be adhered along the extensor digitorum and extensor carpi radialis muscle fibers according to the guidance of the instrument’s guiding program. The average electromyography value will be calculated and used for analysis. Three trials with high signal quality will be selected for each test action.

### 2.8 Patient withdrawal, risks, and benefits

Any participant can withdraw consent at any time during the study, without any consequences. The study will be terminated under the following conditions: new serious disease leading to unstable vital signs, medical complications, hospital discharge, new emotional or psychological problems, and inability to cooperate with the assessment; patients or their families refuse to continue the study. All the potential benefits and risks of the study will be discussed with participants and their caregivers during the initial visits (29).

### 2.9 Adherence reminder sessions

To promote participation and adherence to the protocol, a video of the study introduction will be shown to the potential participants and their caregivers. A study brochure will be given to all the enrolled participants and their caregivers, including brief introduction of the study, calendar reminder of all the important study events, and treatment self-record tables. The participants will also get a information card with the telephone and email contact information of the PI printed on it. For those who do not complete the full study, their data will be used for a further sub-analysis.

### 2.10 Sample size

The primary outcome of this study is FMA-UE. A power analysis of the independent sample t-test was conducted using R package pwr version 1.3.0. According to a previous study, the effect size (d) was set at 0.8, the significance level was 0.05, and the power was 0.85; the calculated sample size was 60 participants in total, 30 participants in each group (43). Based on a dropout rate of 5%, it is estimated that 32 participants need to be recruited in each group.

### 2.11 Data management and confidentiality

Specific tables and forms were designed to collect the research data, including case report forms (CRFs), adverse events (AEs), serious adverse events (SAEs), treatment records, clinical scales and questionnaires. The data will be recorded firstly in paper data collection forms, and then be entered into an electronic database. To maintain data quality and integrity, double entry, valid value, range check, and random recheck and review will be conducted. The study files will be kept by the research group for at least 5 years after the completion of the trial. Only the PI and authorized personnel will have access to the study data. However, the study records may be reviewed by the Medical Ethics Committee of CRRC and relevant scientific research management and regulation institutions. All the group members will be trained to master the SOPs of the intervention and evaluation methods, thus to improve standardization of the study and improve data quality.

The printed paper forms, records, and other materials will be locked in a drawer in the research office. The electrical data will be stored in a password-protected database installed on a laptop. As the data custodian, the PI will hold the key to the drawer, keep the password of the database and laptop. To protect the participants’ privacy, the research team will maintain confidentiality of the paper and electrical trial files. Each participant will be assigned a unique identification (ID) as a secret code; minimum personal data will be collected and kept. The coded participant level data will be described anonymously and will not be shared. All the information that could identify a specific participant will be recorded only on the informed consent forms, including name, resident ID, contact details, and address. The research management departments will monitor and audit the records without violating confidentiality.

### 2.12 Statistical analysis

For continuous variables, Shapiro–Wilk normality tests will be performed to assess their normality. Data following normal distribution will be expressed as mean ± standard deviation (SD), while the non-normal distributed data will be described as median and interquartile range (IQR). If each group’s data conforms to normal distribution and homogeneity of variance, the independent sample *t*-test will be used to compare the inter-group difference, and the paired *t*-test will be used to compare the intra-group difference pre- and post-interventions. If the data does not conform to normal distribution and homogeneity of variance, the non-parametric tests will be used for comparisons between and within groups. The categorical variables will be expressed as frequency and percentage; Chi-square test will be used for comparison between and within groups. Pearson or spearman correlation analysis will be used to test the correlation between variables according to their distributions. The significance level will be set as *α* = 0.05. We will use R version 4.2.3 and RStudio version 2024.04.21 (Posit Software, MA) to conduct the statistical analysis (44).

All the analyses will be conducted based on the Intention-To-Treat principle. For participants who quit the study midway, a subgroup analysis will be conducted with the collected outcome data. After the first 20 participants have finished all the evaluations, an interim analysis will be conducted according to the statistical analysis plan.

### 2.13 Safety and AE reporting

All the safety related events occurring during the study period will be monitored and recorded by the therapists. In addition, AEs and SAEs will be reported to the Medical Ethics Committee of CRRC and relevant scientific research management and regulation institutions, they will be categorized as related/not related and expected/not expected.

To ensure patient safety, before each session, the therapist will confirm with the participant and their caregivers that none of the following problems existed: bad sleep, alcohol or drugs use, emotional stress or anxiety, cold, flu, headaches, and migraines. During the interventions or evaluations, the therapist will monitor and record the following potential side effects and discomfort caused by VR or ES: seizures; dizziness; nausea; lightheadedness; motion sickness; loss of awareness; eye strain or muscle twitching; blackouts triggered by light flashes or patterns; involuntary movements; altered, blurred, double vision, or other visual abnormalities; disorientation; excessive sweating; increased salivation; and tiredness or exhaustion.

The intervention or assessment will be immediately discontinued if any of the above symptoms is experienced. After each session of intervention and evaluation, the therapist will survey if the following possible side effects or discomforts occur: visual disturbance; visual and muscular fatigue; upper extremity or neck pain, or musculoskeletal discomfort; VR motion sickness; claustrophobia; cybersickness; nausea; soreness; dizziness; headache; and hypertonicity (45).

### 2.14 Dissemination

The findings of this study will be published in peer-reviewed scientific journals and presented at national or international academic conferences.

## 3 Discussion

To solve the challenging problems caused by upper extremity and hand motor function impairments after stroke, it is urgent to develop new rehabilitation options. By combining established rehabilitation assessments and treatments, new options are available to promote the use of MNS-related rehabilitation strategies. The objective of this study is to explore the clinical efficacy and underlying neuromuscular control mechanisms of VRAO+NMES, thus to provide a theoretical basis and clinical evidence to promote this new intervention’s application in stroke rehabilitation. The combination of central and peripheral stimulation based on VRAO+NMES is innovative and aligns with emerging trends in neurorehabilitation.

According to evidence-based research results, MNS-based rehabilitation strategies have huge potential in clinical practice, especially for patients with limited abilities to execute actions actively. As a new kind of treatment based on MNS theory and VR studies, VRAO+NMES has the potential to achieve better motor function recovery, brain structural and functional plasticity. If the efficacy and mechanism thereof can be confirmed, the VRAO+NMES rehabilitative intervention may be a promising add-on treatment to conventional or standard physiotherapy. What’s more, AOT has been used to improve motor and cognitive outcomes in older adults with mild cognitive impairment (46). In the future, we will test its effects in stroke patients with cognitive impairments and speech dysfunction, to maximize the application of this innovative therapy.

This study intends to use concurrent application of AOT and NMES to stimulate the brain regions and muscles simultaneously. We aim to verify the feasibility of central-peripheral synchronous interventions and its impact on neural pathways related to motor control. A study reported that AOT could promote MNS’s activation in patients with stroke, its effects on corticospinal tract (CST) and MUs’ recruitment have also been reported (15). Our previous study showed that AOT was helpful to improve upper extremity motor functions, ADLs, and motor evoked potential (MEP) amplitude in stroke patients, the neural mechanisms may be the enhanced activation of MNS and CST (47). In another study, we tested the application of swallowing AOT in stroke patients and the potential brain network mechanism using fMRI, which also has positive results (20). Additionally, a recently published pilot study showed that 10 min of single concurrent applications of AO, MI, and PNS had a positive immediate effect on hand dexterity in sub-acute stroke patients (24). These published results provided theoretical and practical basis for us to carry out this study.

As a promising technology, VR has great potential in rehabilitation assessments and interventions (48). Based on computer technology, VR generates a digital environment that is highly similar to the real environment, giving the participants a near-real feeling (49). Common VR presentation devices include computer screens, headsets, and tablets; patients can interact with the VR environment via a mouse, keyboard, and joystick (50). In addition, it is possible to record motor performance parameters during VR rehabilitation training, such as speed, acceleration, and force (51).

An important study trend is the potential use of VR in MNS-based rehabilitation treatments, especially in early neurorehabilitation or patients with severe motor deficits who cannot take part in voluntary therapeutic exercises (52). During AO or synchronous AO+MI, patients need to perceive and comprehend the observed actions, which include motor related cognitive processes. These treatments require attention, concentration, memory, language, problem solving, and executive functions, which all influence the learning process and clinical efficacy (12). What’s more, compared with 2D video observation, the cognitive load of using a VR headset is relatively high; participants need to maintain concentration and keep cognitive effort to view the videos during the interventions and assessments. Therefore, a key factor to influence the design of VR rehabilitation programs is to keep the patient attentive in an engaging and tailored environment during the VR training (53).

The findings of the mechanism sub-study part of this research will provide evidence for the increased neural activity induced by synchronous central and peripheral stimulations, thus to support a variety of interventions aimed at carrying out stimulations on both the central and peripheral levels. Some published studies supported this theory. For example, a study concluded that TMS+NMES could enhance the activities of primary motor cortical networks and sensorimotor peripheral circuits (54). In another study, MI+ES showed excellent effectiveness to improve upper extremity motor function in patients with severe paralysis (55). What’s more, as objective quantitative indicators, fNIRS and sEMG test results may act as good biomarkers of brain and muscle activations. These results can be used to predict rehabilitation outcomes, set realistic rehabilitation goals, confirm optimal treatment time window, choose best training intensity or dosage, and select suitable candidates for this new intervention.

Compared with traditional 2D-video AOT, 360° videos based VRAO+NMES can improve the motivation and engagement level of participants. Compared with one-on-one or face-to-face AOT, VRAO+NMES is more efficient in saving therapists’ time and treatment resources. Based on its technical characteristics, VRAO+NMES can be easily modified to be used in group training and tele-rehabilitation, thus to further improve treatment efficiency and expand user settings (56). The 360° VR videos based tele-rehabilitation has potential to improve psychological state and provide enjoyment for community-dwelling individuals (16). These are all contributing factors to promote popularization and application of VRAO+NMES in community and home settings in combination with conventional therapy. In the future, a cost-effectiveness analysis should be conducted to evaluate the economic benefits of this novel rehabilitative approach, especially its potential application in tele-rehabilitation and home rehabilitation (57). In terms of continuous improvement in treatment, immersive quality, visual representation, interactivity, haptic feedback, audio quality, and the display of the VR contents are key elements to improve VRAO+NMES (13). In addition, artificial intelligence (AI) has evolved significantly in recent years, it is possible to further improve the VRAO+NMES application (58).

There are some limitations of this study. First, this is a single-center study, the interpatient variability may affect interpretation of the study results to generalize the results thereof. To further confirm the study conclusions, more multi-center RCTs with larger sample size should be conducted in the future. Second, due to the nature of physiotherapy, it is not possible to blind the patients and therapists, which may lead to some bias. Third, the follow-up time of this study is limited; a longer follow-up should be conducted to compare the long-term efficacy and lasting duration of effects. Fourth, stroke patients are mainly older adults; using the VR headset or glasses may cause discomfort in a number of patients. It is necessary to develop new VR presentation methods, and explore the difference of 360° videos VR and animated VR (59). Fifth, in the brain imaging study section, due to the limited number of optodes, only activation of unilateral MNS can be tested. Devices with more optodes should be used to study more brain areas and the relationships therein. For example, the change of the functional relationships of MNS and salience network (60). In addition, for other brain regions out of the traditional MNS areas that could be also involved in the observation/execution matching system, but cannot be detected by fNIRS, such as the limbic, cerebellum, and brainstem regions, it is necessary to conduct fMRI studies to provide a deeper understanding (61).

While fNIRS has lower spatial resolution compared to fMRI, it is still sufficient to investigate cortical activity, particularly in regions close to the scalp (e.g., prefrontal cortex, motor cortex and the MNS) (62). Our previous studies has confirmed that fNIRS can be used to detect MNS activations during AO, action imitation and motor execution (11, 21). Unlike fMRI, which requires participants to remain stationary inside a confined scanner, fNIRS is more tolerant of participant movement, allows for more ecologically valid experiments (63). In addition, fNIRS is less intimidating and more comfortable for participants, as it does not involve loud noises or confined spaces (64). What’s more, fNIRS can be used in conjunction with VR headsets and does not require participants to lie down in a scanner, allows for a more seamless integration of brain imaging with immersive experiences (65). In general, fNIRS provides a unique combination of portability, comfort, and temporal resolution that is better aligned with the goals and constraints of our study. This makes fNIRS a more appropriate and effective tool for our research.

## 4 Conclusion

The results of this study will provide evidence for the feasibility and potential clinical efficacy of a new innovative rehabilitative approach VRAO+NMES in stroke rehabilitation. This will promote its clinical applicability and generalize the use of this potentially promising approach in hospital, community and home rehabilitation settings. The fNIRS and sEMG sub-study results may provide biomarkers to predict motor function recovery and rehabilitation outcomes after stroke. These biomarkers may also be helpful to screen patients who are suitable to use this new kind of rehabilitation intervention, thus to help to select best candidates.
